# New Insights into the Molecular Epidemiology and Population Genetics of *Schistosoma mansoni* in Ugandan Pre-school Children and Mothers

**DOI:** 10.1371/journal.pntd.0002561

**Published:** 2013-12-12

**Authors:** Martha Betson, Jose C. Sousa-Figueiredo, Narcis B. Kabatereine, J. Russell Stothard

**Affiliations:** 1 Department of Production and Population Health, The Royal Veterinary College, Hatfield, Hertfordshire, United Kingdom; 2 Parasitology Department, Liverpool School of Tropical Medicine, Liverpool, United Kingdom; 3 Department of Infectious and Tropical Diseases, London School of Hygiene and Tropical Medicine, London, United Kingdom; 4 Vector Control Division, Ministry of Health, Kampala, Uganda; Imperial College London, Faculty of Medicine, School of Public Health, United Kingdom

## Abstract

Significant numbers of pre-school children are infected with S*chistosoma mansoni* in sub-Saharan Africa and are likely to play a role in parasite transmission. However, they are currently excluded from control programmes. Molecular phylogenetic studies have provided insights into the evolutionary origins and transmission dynamics of *S. mansoni*, but there has been no research into schistosome molecular epidemiology in pre-school children. Here, we investigated the genetic diversity and population structure of *S. mansoni* in pre-school children and mothers living in lakeshore communities in Uganda and monitored for changes over time after praziquantel treatment. Parasites were sampled from children (<6 years) and mothers enrolled in the longitudinal Schistosomiasis Mothers and Infants Study at baseline and at 6-, 12- and 18-month follow-up surveys. 1347 parasites from 35 mothers and 45 children were genotyped by direct sequencing of the cytochrome c oxidase (*cox*1) gene. The *cox*1 region was highly diverse with over 230 unique sequences identified. Parasite populations were genetically differentiated between lakes and non-synonymous mutations were more diverse at Lake Victoria than Lake Albert. Surprisingly, parasite populations sampled from children showed a similar genetic diversity to those sampled from mothers, pointing towards a non-linear relationship between duration of exposure and accumulation of parasite diversity. The genetic diversity six months after praziquantel treatment was similar to pre-treatment diversity. Our results confirm the substantial genetic diversity of *S. mansoni* in East Africa and provide significant insights into transmission dynamics within young children and mothers, important information for schistosomiasis control programmes.

## Author Summary

Many pre-school children in sub-Saharan Africa are infected with the parasite *Schistosoma mansoni*, which causes intestinal schistosomiasis. However, there has been no work published on the molecular epidemiology of *Schistosoma* in children under six or the role that these children play in parasite transmission. We analysed the genetic structure of parasite populations collected from mothers and young children living on the shores of Lakes Albert and Victoria in Uganda. Parasite populations were different at the two lakes indicating that there is little flow of parasite genes between the lakes. We were surprised to discover a large amount of genetic variation in parasites sampled from both children and mothers, suggesting that genetic variation is not directly related to duration of exposure to infested water. In addition, we found some evidence that young children are involved in *S. mansoni* transmission. The substantial genetic variation of *S. mansoni* in young children suggests that these parasites could be operating as a source of a variety of genetic traits, including drug susceptibility. Overall our findings offer significant insights into population genetics of *S. mansoni* in pre-school children and their mothers and provide important information for effective control of intestinal schistosomiasis.

## Introduction

The neglected tropical disease schistosomiasis, remains a significant public health problem, particularly in sub-Saharan Africa (SSA), and is caused by infection with parasites of the genus *Schistosoma* [1]. In SSA *Schistosoma mansoni* is responsible for intestinal schistosomiasis and is associated with pathologies ranging from diarrhoea and anaemia to hepatosplenomegaly and portal hypertension [2]. Although *S. mansoni* infects tens of millions of individuals across SSA, its distribution is heterogeneous as its life-cycle depends on water contact, poor sanitation and the presence of suitable habitats for the intermediate host, freshwater snails of the genus *Biomphalaria* [3], [4].

National Control Programmes for schistosomiasis have been established in a number of African countries [5]. These programmes are based on regular mass distribution of the anthelminthic drug praziquantel to school-aged children and their main aim is control of morbidity. School-aged children have been particularly targeted because they usually show the highest infection intensities. However, recent work has demonstrated that infants and pre-school children are also at high risk of infection and can benefit from praziquantel treatment [6], [7]. In 2012, new targets were set by WHO calling for elimination of schistosomiasis in certain African countries by 2020 [8]. To reach these goals, it will be necessary to implement measures such as snail control and health education in addition to preventive chemotherapy, and to target the whole community including pre-school children [1].

The molecular evolution and phylogenetics of *Schistosoma* species of medical and veterinary importance have been studied in some detail using mitochondrial and nuclear markers [9]–[13]. In addition, efforts have been made to dissect the genetic diversity and population structure of individual *Schistosoma* species. DNA barcoding approaches [14]–[16] involving comparison of sequence variations in a portion of cytochrome oxidase I (*cox*1) have been particularly informative [17]–[20]. Analysis of *S. mansoni* samples from across the globe revealed that *S. mansoni* separates geographically into five major lineages [11], [20]. The lower genetic diversity observed in the New World compared to the Old World and clustering of samples from the Americas with those from West Africa suggested that *S. mansoni* was recently introduced into the New World, perhaps with slaves infected with intestinal schistosomiasis from West Africa [11], [20].

Particularly high levels of genetic diversity have been observed in East African *S. mansoni* populations [17], [20]–[23]. Barcoding of *S. mansoni* collected from school-aged children and *Biomphalaria* snails on the shores of Lake Albert (LA) in Uganda and Lake Victoria (LV) in Kenya, Tanzania and Uganda revealed extensive population diversity with genetic differentiation between LA and LV [17], [18]. Interestingly, most parasite diversity was at the level of the individual host rather than at the level of geographical location [17]. Similar levels of within-child diversity have been observed in Kenya using microsatellite markers [22].

To date there have been no studies investigating the genetic structuring of *Schistosoma* populations in pre-school children. It could be hypothesised that because the cumulative exposure and infection window has been shorter for younger children than school-aged children or adults, parasite diversity in pre-school children may be lower than in older individuals. However, heterogeneities in local transmission may also be more manifest in younger children [24]. The aim of this study was to investigate the genetic diversity and population structuring of *S. mansoni* parasites in a cohort of pre-school children and their mothers living in Ugandan lakeshore communities before and after praziquantel treatment, in so doing we hoped to address the role of young children in local parasite transmission.

## Methods

### Ethics statement

The London School of Hygiene and Tropical Medicine (LSHTM 5538.09) and the National Council of Science and Technology, Kampala, Uganda, granted ethical approval for the Schistosomiasis in Mothers and Infants (SIMI) study. Before selection, all families received an information leaflet describing the study objectives and procedures, which were explained in detail by the local Vector Control Division district officer. Informed consent documented by signature or fingerprint (in cases of illiteracy) was obtained from each mother on her own behalf and on behalf of her child or children who were participating in the study. Fingerprint consent procedures were specifically approved by the research ethics committees of the London School of Hygiene and Tropical Medicine and the Ugandan National Council of Science and Technology.

### Epidemiological surveys

Schistosome parasites were collected during the SIMI longitudinal study carried out in six communities on the shores of LA and LV in Uganda [6], [7], [25]. 1856 mothers and children under six were recruited into the study at baseline and followed-up at three, six, 12 and 18 months (LV only) after baseline survey. At each time point parasitological assessment of *S. mansoni* was carried out [25]. At baseline all participants were offered praziquantel (40 mg/Kg) and albendazole (400 mg) according to WHO treatment guidelines [26]. At subsequent surveys, praziquantel was provided on the basis of a positive cathodic-circulating antigen (CCA) test [27]. At each timepoint, malaria treatment (artemether-lumefantrine) was provided to study participants on the basis of a positive malaria rapid diagnostic test (RDT) result (Paracheck or First Response). During each survey mothers were asked a suite of questions on behalf of themselves and their child(ren) pertaining to health-seeking behaviour, water-contact and socio-economic status. Copies of the questionnaire are available on request from the corresponding author. Anaemia and faecal occult blood (FOB) were assessed as reported [25], [28] and clinical examination of liver and spleen pathology was carried out as described [29].

### Sample collection

At baseline schistosome eggs were isolated from the stool of participants who were egg-patent or CCA-positive for *S. mansoni* infection. Particular effort was made to obtain eggs from family groups. During subsequent surveys, eggs were collected from the same individuals sampled at baseline or members of the same family (if *S. mansoni* positive). In addition, for the 6 month survey at Bugoigo a selection of samples for egg isolation was chosen at random from a list of schistosome-positive individuals. To obtain eggs, stool samples were diluted in bottled water and an adaptation of the Pitchford-Visser funnel method was used [18]. After isolation, eggs were exposed to light for several hours to stimulate miracidial hatching. Individual miracidia and/or eggs were harvested under a dissecting microscope and placed on FTA® indicator cards (Whatman). Alternatively miracidia/eggs were placed in wells of a 96-well PCR plate, each well containing 7.5 µl of RNAlater® (Ambion, Life Technologies Ltd). Samples were transported to the UK for molecular analysis.

### DNA extraction and *cox*1 PCR

2.0 mm punches were taken from the centre of each parasite spot on FTA® cards and processed as described [20]. DNA was extracted from samples in RNALater® using the DNAeasy (Qiagen Ltd) [30] or the GeneJet (Fermentas, ThermoFisher Scientific Ltd) genomic DNA extraction kits according to the manufacturers' instructions. A 540 bp fragment was amplified from the FTA punch or 3 µl of genomic DNA using the ASMIT1 [18] and Cox1_Schist_3′ [10] primers and illustra™ puReTaq Ready-To-Go PCR Beads (GE Healthcare). The following cycling conditions were used: 95°C for 1 min, 40 cycles of 95°C for 30 s, 40°C for 30 s and 72°C for 2 min, with a 7 min extension of 72°C. PCR products were cleaned using the QIAquick PCR purification kit (Qiagen Ltd) or SureClean reagent (BioLine Reagents Ltd) according to the manufacturers' instructions. Samples were sequenced on a 3130×l Genetic Analyser (Applied Biosystems) running BigDye v3.1 sequencing chemistry.

### *Cox*1 sequence and phylogenetic analysis

DNA sequences were manually edited using CLC Workbench v6 based on inspection of sequence chromatograms and truncated to the 396 bp ASMIT region for comparison with *cox*1 sequences from previous studies [17], [18], [31]. BLAST was used to search for exact sequence matches in Genbank. Sequences were aligned in MacClade v4.05 and Collapse v1.2 was used to identify samples with identical haplotypes. The sequences of novel haplotypes (H176–H359) were submitted to Genbank (accession numbers KC964660–KC964848). To determine efficiency of schistosome sampling, the cumulative number of unique haplotypes was plotted against sequentially sampled infrapopulations (individual hosts) [32]. Phylogenetic analysis of all identified haplotypes was carried out in MEGA v5 [33]. *Cox*1 sequences representing the five lineages of *S. mansoni* across Africa [20] were included for comparison and an *S. rodhaini cox*1 sequence was used to root trees. Bootstrapping (1000 replicates) was carried out to test branch reliability.

### Population genetic analysis

Sequences from all parasite samples were imported into DNASP v5 [34]. Haplotype diversity (h), overall nucleotide diversity (Π) (with Jukes-Cantor corrections) [35], [36] and nucleotide diversity for synonymous and non-synonymous substitutions were determined for the whole population of parasites sampled, and parasite populations stratified by host type (mother or child), lake, village, survey timepoint and individual host (where ≥6 parasites were barcoded). Genetic diversity in individual hosts/infrapopulations was summarised by host type, lake and survey timepoint so that comparisons could be made with genetic diversity measures based on pooled samples (infrapopulation approach versus component population approach) [23], [37]. As diversity data were not normally distributed, the median was chosen as the measure of central tendency and confidence intervals calculated using the binomial exact method [38]. To take into account the potential relatedness of miracidia within individual hosts, genetic differentiation between parasite populations was analysed using a hierarchical analysis of molecular variance (AMOVA) procedure in Arlequin v3.5.1.2 [23], [39]. Statistical tests of genetic differentiation were carried out using 10000 random permutations. In addition, pairwise analysis of gene flow between parasite infrapopulations was carried out in Arlequin using the Φ_ST_ estimator together with permutation tests (10000 random permutations) of genetic differentiation. The net mean genetic distance [40] between infrapopulations was determined in MEGA and used to draw phylogenetic trees.

### Statistical analysis

For baseline data, statistical associations were investigated (using non-parametric tests) between haplotype and nucleotide diversity in individual hosts (from whom ≥6 parasites were sequenced) and lake, host type (mother or child), host age, infection intensity (number of eggs per gram of stool) and a history of praziquantel treatment (mothers only). To examine associations between morbidity and schistosome diversity, study participants were classified as anaemic if they had a haemoglobin level <11.0 g/dL and FOB positives were categorised as described [25]. Liver and spleen pathology were inferred on the basis of an enlarged liver/spleen and/or a firm/hard liver/spleen consistency [29]. A categorical variable was created to capture overall morbidity, with 0 representing no anaemia, FOB, liver or spleen pathology, 1 representing the presence of one morbidity maker, 2 the presence of two markers and 3 the presence of three/more markers. All statistical analysis was carried out in Stata v11.

### Accession numbers

*S. mansoni cox* 1 haplotypes

V7: FJ750538; V3: FJ750534; A7: FJ750530; H1: GQ415163; H2: GQ415167; H4: GQ415169; H8: GQ415171; H10: GQ415173; H14: GQ415176; H16: GQ415179; H17: GQ415182; H18: GQ415185; H20–H23: GQ415189–92; H29: GQ415200; H31: GQ415202; H35: GQ415208; H36: GQ415211; H38: GQ415215; H41: GQ415218; H42: GQ415220; H46: GQ415227; H47: GQ415228; H50: GQ415231; H53: GQ415234; H54: GQ415235; H60: GQ415242; H63: GQ415245; H65: GQ415247; H67: GQ415249; H75: GQ415258; H77–H79: GQ415261–3; H84: GQ415268; H87: GQ415271; H89: GQ415273; H92: GQ415276; H99: GQ415283; H100: GQ415284; H103: GQ415288; H106: GQ415291; H107: GQ415292; H116: GQ415301; H117: GQ415302; H123: GQ415308; H124: GQ415309; H126: GQ415311; H128: GQ415313; H131: GQ415316; H136: JF290433; H137: HM055378; H140: HQ839768; H146: JF274042; H150: JF274046; H153: JF440336; H155: JF508492; H158: JF508495; H176–H359: KC964660–848 (release date 1 Aug 2013); CA1b: JQ289589; CA1f: JQ289593; CA1h: JQ289595; CK1e: JQ289600; CK1g: JQ289602; CK1h: JQ289603; CK1j: JQ289605; CK1l–CK1n: JQ289607–9; CK1u: JQ289616; EG1: JQ289620; KE1: JQ289618; NI1a: JQ289624; NI1g: JQ289630; NI1h: JQ289631; NI1k: JQ289634; NI1m: JQ289636; NI1p: JQ289639; SA1b: JQ289653; SE2a: JQ289655; SE2c: JQ289657; SE2j: JQ289668; SE3e: JQ289682; SE4c: JQ289690; TA1c: JQ289693; TA1g: JQ289697; TA1h: JQ289698; TA1n: JQ289704; TA1p: JQ289706; UG3a: JQ289716; UG3b: JQ289717; UG4a: JQ289721; UG4e: JQ289725; ZA1b–ZA1j: JQ289728–36.

*S. rodhaini cox*1: JQ314100

## Results

### Haplotypes

**Figure 1:**
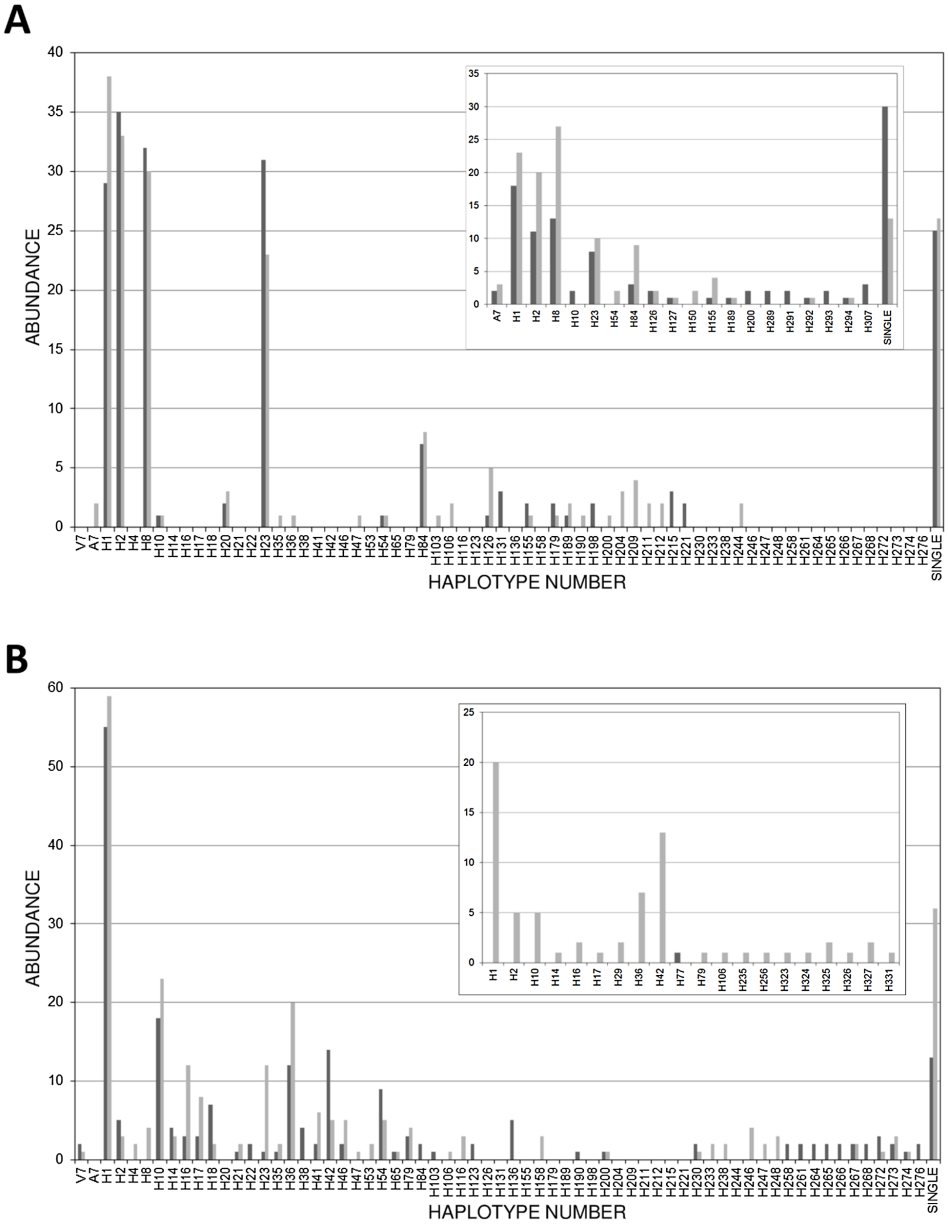
Haplotype abundance barcharts for Lake Albert (A) and Lake Victoria (B). The main barcharts display haplotypes recovered during the baseline surveys and the inset barcharts display haplotypes isolated during the six-month follow up surveys. Dark grey bars indicate haplotypes identified in mothers whereas light grey bars represent haplotypes isolated from children. “Single” refers to unique haplotypes which were only identified once during the surveys.

**Figure 2:**
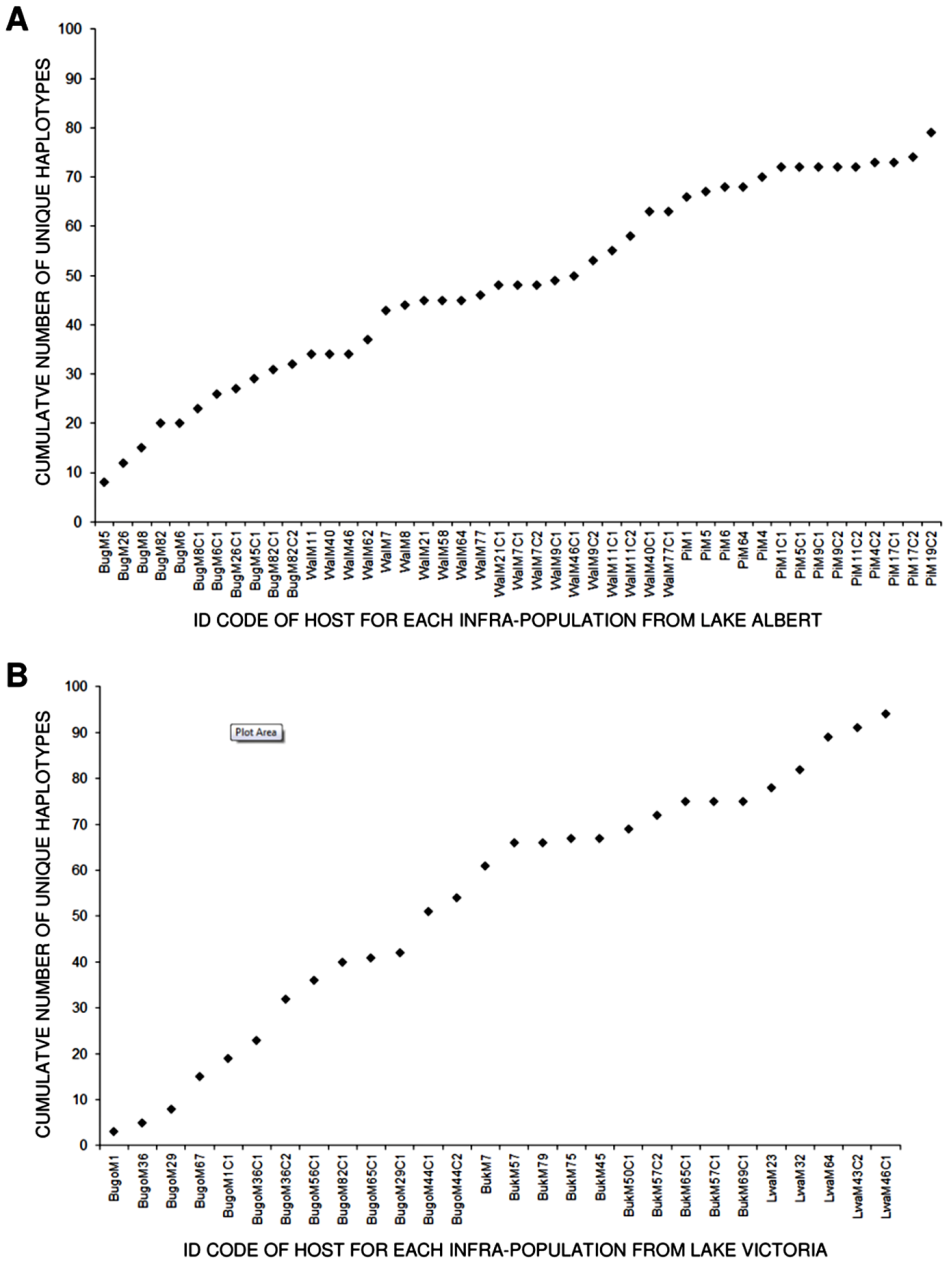
Cumulative number of unique haplotypes identified at baseline plotted against the identification code of each sequentially sampled host from Lake Albert (A) and Lake Victoria (B).

*Schistosoma mansoni cox*1 sequences were obtained from 1347 parasites collected from 35 mothers and 45 children. The *cox*1 region was highly diverse with 246 different haplotypes identified, of which 184 were novel. Figure 1 shows how often each haplotype was identified in parasites collected from mothers and children at baseline and six month follow-up. H1 was very common at both lakes, whereas other haplotypes (e.g. H2, H8, H10 and H23) were more common at one lake rather than the other. In addition, a number of haplotypes were extremely rare, only being identified once during the study. Plotting the cumulative number of unique haplotypes identified against sequentially sampled hosts (each representing a parasite infrapopulation) suggested that the infrapopulations sampled were not fully representative of the *cox*1 haplotype diversity at either lake, as the curves did not approach the asymptote after 36 infrapopulations for LA or 28 infrapopulations for LV (Figure 2). Consistent with this, 70 haplotypes not sampled at baseline were identified in follow-up surveys.

### Phylogenetic analysis

**Figure 3:**
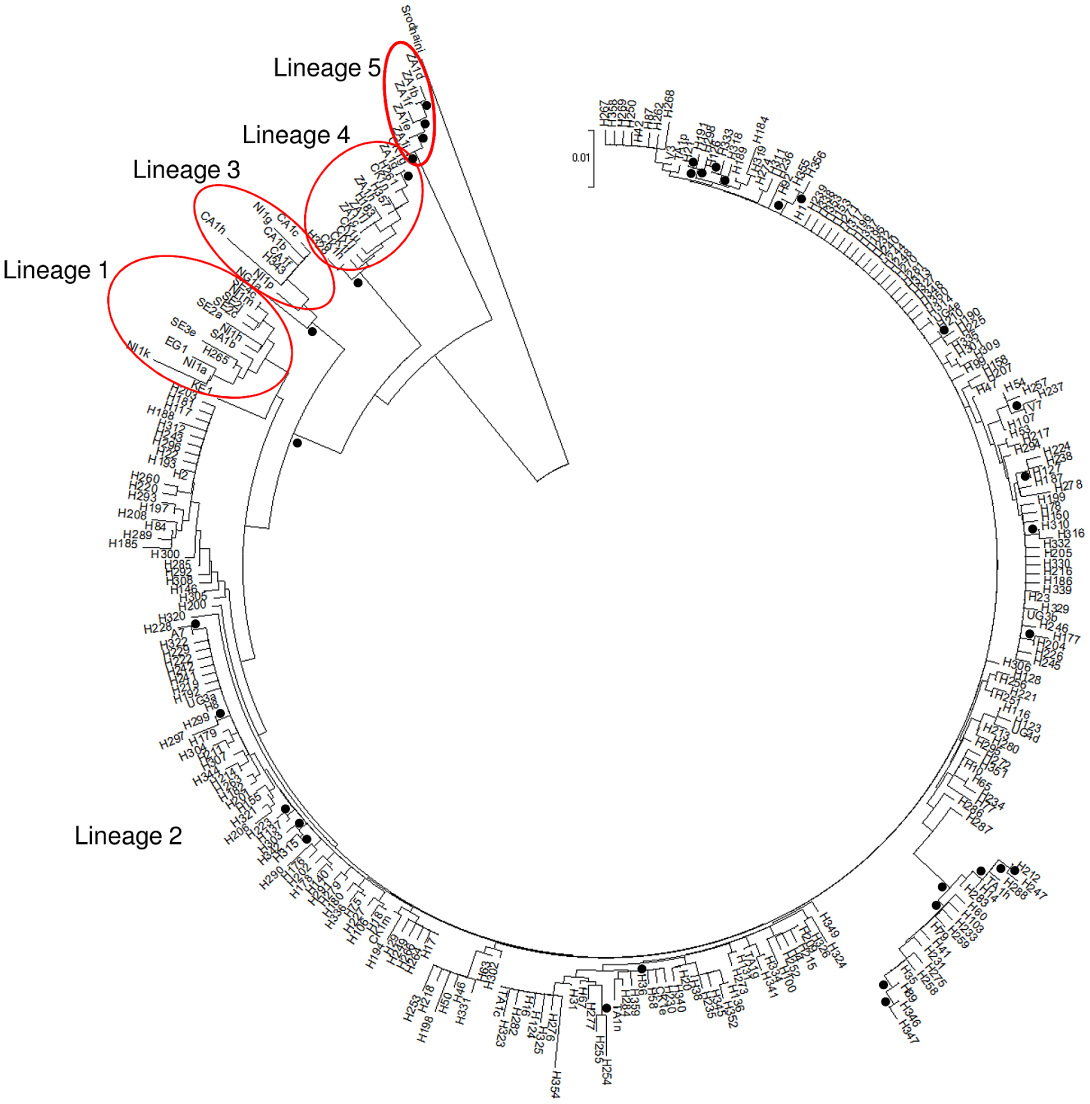
A neighbour joining tree based on Kimura-2-parameters representing phylogenetic relationships between haplotypes identified during the baseline surveys and selected haplotypes from across the globe. Black dots represent branches for which bootstrap support was over 75% (1000 replicates). Most haplotypes fall into lineage 2 as identified by Webster *et al.* [20]. Haplotypes falling into lineages 1, 3, 4 and 5 are indicated using red oval outlines.

Phylogenetic analysis was carried out on all haplotypes isolated in this study and including a selection of *S. mansoni cox*1 haplotypes previously characterised from different geographical regions [20]. The same five lineages were observed as identified by Webster *et al*. (Figure 3). Interestingly, although most of the haplotypes identified in the present study fell into lineage 2 (East Africa), four haplotypes (H183, H261, H328 and H357) were found in lineage 4 (Zambia and Coastal Kenya), one haplotype (H265) in lineage 1 (West Africa, Brazil and Asia) and one (H343) in lineage 3 (Central West Africa and Niger). Very similar tree topologies were generated using the Neighbour-Joining, Maximum Parsimony and Minimum Evolution methods.

### Genetic diversity

High levels of haplotype and nucleotide diversity in the *cox*1 region were found in schistosome parasites sampled at each lake (Table 1) and similar levels of diversity were observed when the data were stratified by village (Table 2). Surprisingly, except for Bugoto village, parasite populations sampled from children were as diverse as those sampled from their mothers (Tables 1 and 2). Parasite populations were also highly diverse in the *cox*1 region at the infrapopulation level and similar levels of diversity were found in parasites sampled from individual mothers and children, whether related or unrelated (Tables S1 and S2). In addition, *S. mansoni* parasites sampled at six, 12 and 18 months were highly diverse (Table 1). When nucleotide diversity at synonymous and non-synonymous sites was compared between lakes, villages and mothers and children, similar levels of diversity at synonymous sites were found in mothers and children and in different locations (Tables 1 and 2). However, the diversity at non-synonymous sites appeared higher at LV than in LA. This was consistent in mothers and children and between villages (Table 2).

**Table 1:** *Cox*1 diversity stratified by host type, lake system and survey timepoint.

Survey	Lake	Host	N_HOSTS_ a	*n* b	*u* c	*h* d	Π^e^	Π (SS)^f^	Π (NS)^g^
Baseline	Albert	Mothers	20	179	41	0.875±0.012	0.00890	0.03580	0.00056
		Children	25	195	51	0.896±0.011	0.00894	0.03542	0.00075
		All	45	374	80	0.886±0.008	0.00892	0.03557	0.00066
	Victoria	Mothers	12	197	50	0.918±0.016	0.00988	0.03648	0.00174
		Children	16	243	68	0.901±0.012	0.00908	0.03270	0.00175
		All	28	440	94	0.912±0.010	0.00946	0.03448	0.00175
	Both	Mothers	32	376	85	0.918±0.009	0.00986	0.03799	0.00119
		Children	41	438	108	0.925±0.008	0.00939	0.03546	0.00131
		All	73	814	161	0.922±0.006	0.00962	0.03674	0.00126
6 months	Albert	Mothers	8	105	48	0.940±0.013	0.00885	0.03343	0.00120
		Children	7	119	27	0.874±0.016	0.00833	0.03387	0.00039
		All	15	224	63	0.907±0.010	0.00856	0.03361	0.00077
	Victoria	Children	4	68	30	0.863±0.027	0.00766	0.02389	0.00259
		All	5	69	20	0.867±0.027	0.00776	0.02410	0.00265
	Both	Mothers	9	106	49	0.941±0.012	0.00892	0.03355	0.00125
		Children	11	187	44	0.899±0.012	0.00854	0.03196	0.00125
		All	20	293	79	0.915±0.009	0.00869	0.03253	0.00126
12 months	Albert	Mothers	2	14	12	0.978±0.035	0.00880	0.03392	0.00096
		Children	5	90	26	0.875±0.018	0.00895	0.03647	0.00052
		All	7	104	34	0.889±0.016	0.00891	0.03604	0.00058
18 months	Victoria	Mothers	2	15	12	0.962±0.040	0.01045	0.04013	0.00134
		Children	5	122	43	0.922±0.013	0.00980	0.03509	0.00202
		All	7	137	49	0.925±0.012	0.00990	0.03577	0.00195

a N_HOSTS_ = number of hosts;

b *n* = number of haplotypes;

c *u* = number of unique haplotypes;

d *h* = haplotype diversity;

e Π = nucleotide diversity;

f Π (SS) = nucleotide diversity at synonymous sites;

g Π (NS) = nucleotide diversity at non-synonymous sites.

**Table 2:** *Cox*1 diversity stratified by host type and village at baseline and 6 months.

Survey	Village	Host	N_HOSTS_ a	*n* b	*u* c	*h* d	Π^e^	Π (SS)^f^	Π (NS)^g^
Baseline	Bugoigo	Mothers	5	61	20	0.870±0.025	0.00946	0.03858	0.00044
		Children	6	54	21	0.909±0.021	0.01011	0.04077	0.00074
		All	11	115	34	0.898±0.013	0.00977	0.03962	0.00058
	Walukuba	Mothers	10	74	21	0.889±0.018	0.00875	0.03524	0.00054
		Children	10	86	26	0.874±0.020	0.00825	0.03181	0.00093
		All	20	160	40	0.879±0.013	0.00845	0.03327	0.00075
	Piida	Mothers	5	44	13	0.852±0.027	0.00833	0.03268	0.00076
		Children	9	55	20	0.900±0.022	0.00906	0.03665	0.00048
		All	14	99	28	0.880±0.017	0.00868	0.03464	0.00061
	Bugoto	Mothers	4	27	15	0.909±0.038	0.01435	0.05819	0.00141
		Children	9	131	48	0.929±0.014	0.00952	0.03280	0.00229
		All	13	158	54	0.928±0.013	0.01047	0.03765	0.00214
	Bukoba	Mothers	5	68	23	0.912±0.022	0.01045	0.03879	0.00172
		Children	5	68	24	0.899±0.026	0.00848	0.03252	0.00105
		All	10	136	34	0.910±0.018	0.00952	0.03583	0.00140
	Lwanika	Mothers	3	102	27	0.876±0.025	0.00803	0.02804	0.00181
		Children	2	44	18	0.888±0.030	0.00844	0.03180	0.00120
		All	5	146	48	0.888±0.020	0.00824	0.02952	0.00163
6 months	Bugoigo	All	5	78	25	0.889±0.019	0.00895	0.03418	0.00110
	Walukuba	All	7	99	34	0.912±0.015	0.00832	0.03332	0.00054
	Piida	All	3	47	19	0.895±0.027	0.00739	0.02875	0.00071
	Bugoto	All	3	47	23	0.847±0.032	0.00798	0.02413	0.00291
	Lwanika	All	1	21	13	0.838±0.067	0.00626	0.02148	0.00153

a N_HOSTS_ = number of hosts;

b *n* = number of haplotypes;

c *u* = number of unique haplotypes;

d *h* = haplotype diversity;

e Π = nucleotide diversity;

f Π (SS) = nucleotide diversity at synonymous sites;

g Π (NS) = nucleotide diversity at non-synonymous sites.

**Figure 4:**
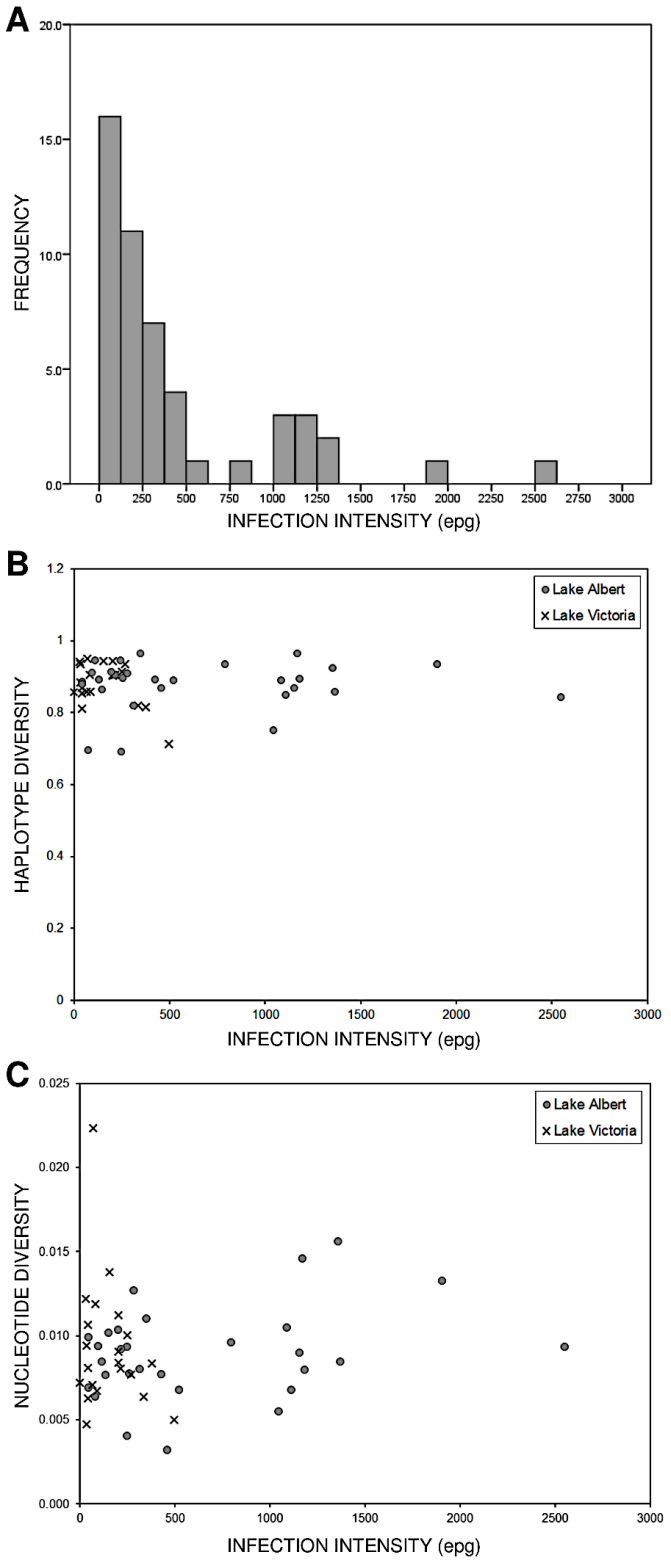
Relationship between infection intensity and genetic diversity at baseline. (A) Histogram displaying typical “overdispersed” distribution of *S. mansoni* infections at baseline. (B) Haplotype diversity of parasites isolated from individual hosts plotted against host infection intensity. (C) Nucleotide diversity of parasites isolated from individual hosts plotted against host infection intensity.

The associations between schistosome *cox*1 diversity and host type (mother or child), lake, history of praziquantel treatment, infection intensity and morbidity were investigated at baseline. There was no evidence for an association between host type, lake and praziquantel treatment history and haplotype or nucleotide diversity (data not shown). Interestingly, nucleotide diversity at non-synonymous sites was associated with lake (Wilcoxon's *W* = 156.5; *p* = 0.004; *N* = 50). There was no correlation between infection intensity and haplotype diversity (Spearman's *ρ* = −0.0287; *p* = 0.834; *N* = 50), or nucleotide diversity (Spearman's *ρ* = 0.1974; *p* = 0.766; *N* = 50) (Figure 4), however nucleotide diversity at non-synonymous sites was negatively correlated with infection intensity (Spearman's *ρ* = −0.3429; *p* = 0.015; *N* = 50). In addition, there was some evidence for a positive correlation between nucleotide diversity at non-synonymous sites and age in children (Spearman's *ρ* = 0.3426; *p* = 0.074; *N* = 28). Finally, no association was observed between any individual morbidity marker and genetic diversity (data not shown), nor was there evidence of an association between overall morbidity and haplotype diversity (*χ* ^2^ = 0.574; *p* = 0.9023; *N* = 36) or nucleotide diversity (*χ* ^2^ = 1.704; *p* = 0.636; *N* = 36).

### Genetic differentiation

**Figure 5:**
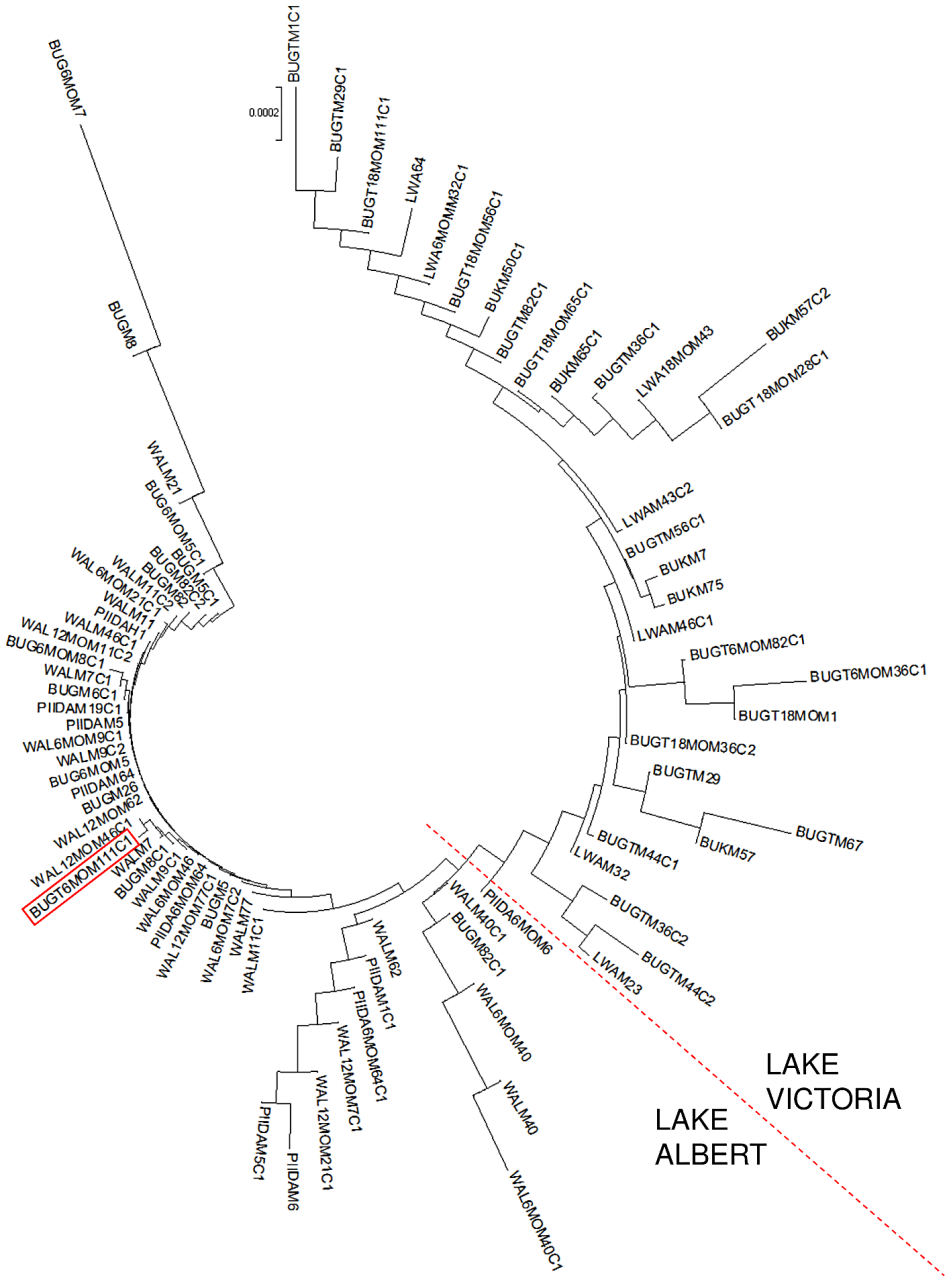
Neighbour joining tree based on the net mean genetic distances between individual infrapopulations sampled at baseline and in follow-up surveys. The red-dotted line indicates the almost-complete segregation between infrapopulations from Lake Albert and those from Lake Victoria. The red rectangle highlights one infrapopulation from Lake Victoria which clusters with infrapopulations from Lake Albert. BUG = Bugoigo; WAL = Walukuba; PIIDA = Piida; BUGT = Bugoto; BUK = Bukoba; LWA = Lwanika. 6 MO = 6 month follow-up; 12 MO = 12 month follow-up; 18 MO = 18 month follow-up. M* = identification code for a mother host; M*C1 or M*C2 = identification code for a child host.

AMOVA analysis, with groups defined based on location or host type, revealed that variation in parasite populations within individual hosts accounted for most of genetic variation observed (Tables 3, 4 and S3). Nevertheless there was evidence of restricted gene flow between parasite populations at LA and LV but not between mothers and children (Tables 3 and 4) or between individual villages at LA or LV (Table S3). Interestingly, there was evidence of genetic differentiation between infrapopulations at LV but not at LA, although this only accounted for ∼3.5% of overall variation (Tables 4 and S3). Comparison of parasite populations sampled at different time points revealed little differentiation between populations collected at baseline and 6 months or 12 months in Bugoigo, Walukuba or Piida (LA), however there was evidence of differentiation between parasites obtained at different timepoints from Bugoto (Table 5). Pairwise analysis of gene flow between infrapopulations revealed little differentiation between infrapopulations in LA villages at baseline or in follow-up surveys (data not shown). For LV, the pattern was more complex with restricted gene flow between some infrapopulations, even within the same village. Phylogenetic analysis revealed that infrapopulations from LA clustered together and those from LV clustered together (Figure 5). Overall the genetic distances between infrapopulations from LV were larger than between infrapopulations from LA. However, there was no clustering of infrapopulations by survey timepoint.

**Table 3:** AMOVA results for mothers and children showing evidence of genetic differentiation between lake systems at baseline.

	Mothers					Children				
Source of variation	Sum of squares	Variance components	% of variation	Φ_ST_ a	*P* value^b^	Sum of squares	Variance components	% of variation	Φ_ST_	*P* value
Between lakes	34.7	0.171	8.4	0.0845	<0.0001	34.2	0.148	7.7	0.0768	<0.0001
Among hosts within lake regions	69.5	0.049	2.3	0.1071	0.022	81.97	0.035	1.8	0.0950	0.037
Within hosts	620.5	1.804	89.3	0.0248	<0.0001	690.1	1.697	90.5	0.0197	<0.0001

a Φ_ST_ estimator of genetic differentiation;

b *P* value from permutation test of genetic differentiation (10000 permutations).

**Table 4:** AMOVA results for two lake systems showing little evidence of genetic differentiation between mothers and children at baseline.

	Lake Albert					Lake Victoria				
Source of variation	Sum of squares	Variance components	% of variation	Φ_ST_ a	*P* value^b^	Sum of squares	Variance components	% of variation	Φ_ST_	*P* value
Between mothers & children	1.2	−0.004	−0.2	0.0000	0.671	4.3	0.004	0.2	0.0023	0.242
Among hosts within mother or child populations	79.7	0.014	0.8	0.0000	0.182	71.7	0.064	3.5	0.0368	0.0004
Within hosts	572.6	1.740	99.4	0.0000	0.294	738.0	1.791	96.3	0.0345	<0.0001

a Φ_ST_ estimator of genetic differentiation;

b *P* value from permutation test of genetic differentiation (10000 permutations).

**Table 5:** Genetic differentiation between surveys assessed by hierarchical AMOVA.

		Baseline & 6 months		Baseline & 12 or 18 months^a^		6 months & 12 or 18 months^a^	
		Φ_ST_ b	*P* value^c^	Φ_ST_	*P* value	Φ_ST_	*P* value
Bugoigo	Mothers	0.0000	0.513	-	-	-	-
	Children	0.0047	0.429	-	-	-	-
Walukuba	Mothers	0.0000	0.595	0.0000	0.816	0.0000	0.754
	Children	0.0000	0.777	0.0075	0.279	0.0071	0.393
Piida	Mothers	0.0000	0.529	-	-	-	-
	Children	0.0000	0.502	-	-	-	-
Bugoto	Mothers	-	-	0.0085	0.405	-	-
	Children	0.0491	0.005	0.0020	0.242	0.0417	0.012

a 12 months for Walukuba and 18 months for Bugoto;

b Φ_ST_ estimator of genetic differentiation;

c *P* value from permutation test of genetic differentiation (10000 permutations).

## Discussion

This is the first study to investigate the population genetics of *S. mansoni* in pre-school children. We found similar high levels of genetic diversity in pre-school children and their mothers, both at baseline and after praziquantel treatment. Overall diversity was comparable between LA and LV and between individual villages. However, nucleotide diversity at non-synonymous sites was significantly higher in LV than LA. There was also evidence of genetic differentiation between parasite populations at the two lakes.

Our results are consistent with a number of studies demonstrating that *S. mansoni* is highly diverse in East Africa [11], [20], [23]. Interestingly, despite barcoding over 1340 individual parasites and identifying 184 novel haplotypes, it appears that our sampling did not capture the full *cox*1 diversity at either lake (Figure 2). Although this could be considered a limitation of our study, it is consistent with the fact that new *cox*1 haplotypes are identified every time *S. mansoni* is sampled from East Africa [17], [18], [20], [31], [41]. Based on mathematical models of microsatellite data, French *et al*. have suggested that sampling more hosts (infrapopulations) rather than more miracidia per host leads to more robust estimates of parasite population diversity [37]. It is important to have effective sampling strategies in place for research and monitoring and evaluation of control programmes, however given the level of variation which we and others have observed, it is likely to be very difficult to obtain a truly representative sample of *S. mansoni* diversity in East Africa.

Our observation that genetic diversity is similar in mothers and young children is somewhat unexpected. Diversity in *Schistosoma* infrapopulations could be anticipated to depend on the degree of host exposure to genetically diverse parasites in the environment and on parasite interaction with the host immune system. Our findings contradict the hypothesis, based on a trickle model of infection [42], [43], that young children would show lower *S. mansoni* genetic diversity than mothers due to a shorter cumulative exposure window. Using small GPS dataloggers, we have demonstrated that young children come into contact with water at the margins of Lake Albert for around 30 minutes each day [44]. It is possible that the trickle dynamic becomes rapidly saturated, i.e. the majority of exposures result in infection in the first five minutes and not much longer, with subsequent acquisition of new genotypes as they emerge from snails. In addition, we have shown that young children can become infected with *S. mansoni* from six months of age [45]. The fact that by two years of age some children had acquired highly diverse infections suggests that a cumulative exposure window of up to 1.5 years is sufficient. Although we did not observe a correlation between infection intensity and parasite diversity, which is somewhat counter-intuitive, infections must be egg patent for successful harvesting of eggs using the Pitchford-Visser method, which may bias sampling towards individuals with a higher burden of infection [46]. Alternatively, the sample size (50 infrapopulations) may not have been large enough to detect a correlation between infection intensity and diversity. Interestingly, there was some suggestion of a positive correlation between host age and *cox*1 diversity at non-synonymous sites in children, potentially because of the more developed immune system in older children. Diversifying selection by host immune systems is an important explanation for pathogen antigenic variation [47] and there is evidence that host immunity drives genetic diversity in (male) schistosomes [48]. Since *cox*1 encodes an intracellular protein, it is not exposed to the immune system in intact schistosomes, but it is conceivable that particular *cox*1 haplotypes are associated with specific polymorphisms in antigens under selection pressure. As a mitochondrial gene, *cox*1 variation is only related to the diversity of female worms, but it is possible that host immunity also drives diversity in female schistosomes. Inspection of genetic variation within genes whose products are under immune-surveillance would be informative.

Consistent with previous work, we found evidence for genetic differentiation between *S. mansoni* populations at LA and LV [18]. This is unsurprising given that the field sites on the two lakes are over 300 km apart and that, based on questionnaire data, there was little evidence of individuals moving between the two lakes. Furthermore, human populations are different on the two lakes, with individuals on LA belonging mainly to the Bugungu and Alur tribes and those on LV belonging to the Busoga people. Interestingly, *cox*1 diversity at non-synonymous sites was higher at LV than LA, which may reflect differences in diversifying selection caused by host immunity at the two lakes. The snail intermediate host may also play a role, since the composition of *Biomphalaria* snail populations is different at the two lakes, with *B. stanleyi* found only at LA, *B. choanomphala* only at LV and *B. sudanica* and *B. pfeifferi* at both lakes [49]–[52]. Variations in *S. mansoni*-associated morbidity have been observed between LA and LV which may be due, at least in part, to genetic differentiation between schistosome populations, leading to somewhat different host pathologies [7], [25], [53].

Intriguingly, whereas there was little genetic structuring between parasite populations in individual hosts at LA, there was genetic differentiation between infrapopulations at LV. This could reflect the fact that the lake shore of LV is more convoluted than that of LA, providing micro-environments for transmission. Such micro-environments could mean that even individuals living in the same village are exposed to different parasite populations, depending on where and when they come into contact with infested water. In all villages there was generally little evidence of genetic differentiation between parasite infrapopulations from hosts belonging to the same family. The transmission dynamics of *S. mansoni* are substantially different at LA and LV: overall infection prevalence at baseline was higher at LA than LV and individuals were more rapidly re-infected at LA [6], [7], which could also influence parasite populations.

Similar levels of *S. mansoni* genetic diversity were observed at the lake, village and host level in follow-up surveys after praziquantel treatment, suggesting that six months is sufficient for acquisition of diverse infections and that therapy did not impact on the host immune system to reduce diversity. There was generally little genetic differentiation between parasite populations pre- and post-treatment at LA. At LV schistosome populations did show different genetic structures at baseline and six months, although this could be because parasites were successfully sampled from only three children at six months compared with nine at baseline. When parasite populations from the same hosts were compared between surveys, there was generally little evidence of genetic differentiation between surveys with infrapopulations clustering by lake rather than timepoint. Overall these results suggest that parasite population structure in the lake-shore communities was relatively stable over the study period. Although we did not directly test the clearance of parasites after praziquantel treatment in the present study, we have recently published data from the same villages demonstrating that cure rates were low in very young children and those with a history of previous praziquantel treatment [54]. Thus, it is also possible our findings could represent non-clearance of parasites as well as or rather than reinfection from a pool of parasites with a stable population structure. In contrast to our results, a reduction in genetic diversity in *S. mansoni* sampled from Tanzanian school children was observed after one round of treatment and genetic differentiation between pre- and post-treatment populations [55]. These differing findings may reflect the fact that only a subset of mothers and children in the communities were treated during our study in contrast to most school children in the Tanzanian study. In addition, different molecular markers were used and differences in ecology, transmission dynamics and water contact patterns are likely.

It is now evident that pre-school children can become infected with *S. mansoni* at an early age [6], but their role in *S. mansoni* transmission is still somewhat unclear. A comparison of *cox*1 haplotypes identified only in parasites from pre-school children with haplotypes discovered in cercariae shed by *Biomphalaria* [17], [31] has revealed two haplotypes (H150 at LA and H29 at LV) which are found in both pre-school children and snails. This provides circumstantial evidence that young children play a role in *S. mansoni* transmission but further work is required to confirm this. If true, the high genetic diversity of *S. mansoni* in young children suggests that they could be currently operating as local refugia of meta-populations, providing pools of susceptible genes to dilute genes conferring praziquantel tolerance selected in treated populations (i.e. school children) [56]. High diversity also means that parasites in pre-school children may act as a source of other genetic traits and highlights the need to monitor praziquantel effectiveness and changes in parasite population structure in pre-school children once treatment begins.

To conclude, this study provides novel insights into the epidemiology, genetic diversity and population dynamics of *S. mansoni* in young children and mothers in Uganda, important information for effective ongoing control of intestinal schistosomiasis.

## Supporting Information

Table S1 ***Cox*** **1 diversity stratified by individual host.**
(DOC)

Table S2 ***Cox*** **1 diversity in infrapopulations summarised by host type, lake system and survey timepoint.**
(DOC)

Table S3 **AMOVA results showing little evidence of genetic differentiation between villages at Lake Albert or Lake Victoria at baseline.**
(DOC)
