# Supplementary material for: New Insights into the Molecular Epidemiology and Population Genetics of Schistosoma mansoni in Ugandan Pre-school Children and Mothers
Source: PLoS Negl Trop Dis. 2013 Dec 12;7(12):e2561. doi: 10.1371/journal.pntd.0002561 (PMC3861247; doi:10.1371/journal.pntd.0002561)
Supplement: Table S1 — Cox 1 diversity stratified by individual host. (DOC) [file pntd.0002561.s001.doc]

**Table S1. *Cox*1 diversity stratified by individual host**

| Village | ID^a^ | Age (y)^b^ | Survey | *n*^c^ | *u*^d^ | *h*^e^ | Π^f^ | Survey | *n* | *u* | *h* | Π |
| --- | --- | --- | --- | --- | --- | --- | --- | --- | --- | --- | --- | --- |
| Bugoigo | M5^g^ | 47 | Base^h^ | 18 | 8 | 0.843 ± 0.060 | 0.00936 | 6 mo^i^ | 16 | 11 | 0.933 ± 0.048 | 0.00919 |
|  | M5C1 | 3 | Base | 9 | 6 | 0.889 ± 0.091 | 0.01048 | 6 mo | 30 | 10 | 0.830 ± 0.030 | 0.00840 |
|  | M6C1 | 3 | Base | 9 | 7 | 0.944 ± 0.070 | 0.00934 | - | - | - | - | - |
|  | M7 | 33 | - | - | - | - | - | 6 mo | 15 | 10 | 0.857 ± 0.090 | 0.00917 |
|  | M8 | 35 | Base | 13 | 8 | 0.885 ± 0.070 | 0.00993 | - | - | - | - | - |
|  | M8C1 | 5 | Base | 12 | 8 | 0.942 ± 0.057 | 0.01563 | 6 mo | 14 | 8 | 0.769 ± 0.083 | 0.00725 |
|  | M26 | 23 | Base | 10 | 7 | 0.933 ± 0.062 | 0.00962 | - | - | - | - | - |
|  | M82 | 37 | Base | 18 | 15 | 0.863 ± 0.061 | 0.01019 | - | - | - | - | - |
|  | M82C1 | 4 | Base | 12 | 7 | 0.879 ± 0.075 | 0.00690 | - | - | - | - | - |
|  | M82C2 | 2 | Base | 8 | 5 | 0.857 ± 0.108 | 0.00846 | - | - | - | - | - |
| Walukuba | M7 | 40 | Base | 14 | 8 | 0.912 ± 0.049 | 0.01036 | - | - | - | - | - |
|  | M7C1 | 2 | Base | 17 | 4 | 0.750 ± 0.058 | 0.00550 | 12 mo^j^ | 13 | 6 | 0.872 ± 0.054 | 0.00882 |
|  | M7C2 | 3 | - | - | - | - | - | 6 mo | 11 | 6 | 0.855 ± 0.085 | 0.00777 |
|  | M9C1 | 4 | Base | 6 | 5 | 0.933 ± 0.122 | 0.01328 | 6 mo | 16 | 9 | 0.925 ± 0.039 | 0.01050 |
|  | M9C2 | 2 | Base | 16 | 8 | 0.892 ± 0.048 | 0.00769 | - | - | - | - | - |
|  | M11 | 35 | Base | 12 | 6 | 0.909 ± 0.046 | 0.01270 | - | - | - | - | - |
|  | M11C1 | 5 | Base | 12 | 8 | 0.894 ± 0.078 | 0.00768 | - | - | - | - | - |
|  | M11C2 | 3 | Base | 8 | 7 | 0.964 ± 0.077 | 0.01461 | 12 mo | 9 | 5 | 0.861 ± 0.087 | 0.00777 |
|  | M21 | 32 | Base | 7 | 5 | 0.905 ± 0.103 | 0.00922 | - | - | - | - | - |
|  | M21C1 | 5 | 6 mo | 24 | 11 | 0.906 ± 0.032 | 0.00811 | 12 mo | 30 | 10 | 0.828 ± 0.052 | 0.00891 |
|  | M40 | 32 | Base | 6 | 4 | 0.867 ± 0.129 | 0.00321 | 6 mo | 19 | 11 | 0.930 ± 0.034 | 0.00799 |
|  | M40C1 | 5 | Base | 11 | 8 | 0.891 ± 0.092 | 0.00768 | 6 mo | 9 | 4 | 0.806 ± 0.089 | 0.00366 |
|  | M46 | 25 | - | - | - | - | - | 6 mo | 18 | 12 | 0.935 ± 0.041 | 0.00827 |
|  | M46C1 | 5 | Base | 9 | 7 | 0.944 ± 0.070 | 0.00848 | 12 mo | 14 | 10 | 0.932 ± 0.060 | 0.00729 |
|  | M62 | 35 | Base | 17 | 9 | 0.890 ± 0.049 | 0.00677 | 12 mo | 12 | 11 | 0.985 ± 0.040 | 0.00964 |
|  | M77 | 28 | Base | 12 | 5 | 0.848 ± 0.059 | 0.0068 | - | - | - | - | - |
|  | M77C1 | 2 | - | - | - | - | - | 12 mo | 24 | 12 | 0.909 ± 0.033 | 0.01009 |
| Piida | M1 | 30 | Base | 8 | 7 | 0.964 ± 0.077 | 0.01101 | - | - | - | - | - |
|  | M1C1 | 4 | Base | 13 | 8 | 0.910 ± 0.056 | 0.00940 | - | - | - | - | - |
|  | M5 | 35 | Base | 15 | 5 | 0.819 ± 0.052 | 0.00800 | - | - | - | - | - |
|  | M5C1 | 4 | Base | 9 | 4 | 0.694 ± 0.147 | 0.00640 | - | - | - | - | - |
|  | M6 | 27 | Base | 11 | 4 | 0.691 ± 0.128 | 0.00406 | 6 mo | 11 | 8 | 0.927 ± 0.066 | 0.00684 |
|  | M19C1 | 4 | Base | 22 | 10 | 0.896 ± 0.037 | 0.00777 | - | - | - | - | - |
|  | M64 | 20 | Base | 6 | 4 | 0.867 ± 0.129 | 0.00900 | 6 mo | 21 | 11 | 0.919 ± 0.034 | 0.00760 |
|  | M64C1 | 5 | - | - | - | - | - | 6 mo | 15 | 6 | 0.790 ± 0.079 | 0.00747 |
| Bugoto | M1 | 30 | - | - | - | - | - | 18 mo^k^ | 6 | 6 | 1.000 ± 0.096 | 0.00847 |
|  | M1C1 | 3 | Base | 6 | 5 | 0.933 ± 0.122 | 0.00473 | - | - | - | - | - |
|  | M28C1 | 5 | - | - | - | - | - | 18 mo | 11 | 7 | 0.927 ± 0.054 | 0.01105 |
|  | M29 | 26 | Base | 7 | 5 | 0.905 ± 0.103 | 0.00802 | - | - | - | - | - |
|  | M29C1 | 5 | Base | 8 | 5 | 0.857 ± 0.108 | 0.00671 | - | - | - | - | - |
|  | M36C1 | 5 | Base | 15 | 9 | 0.905 ± 0.054 | 0.00836 | 6 mo | 17 | 6 | 0.735 ± 0.077 | 0.00602 |
|  | M36C2 | 4 | Base | 25 | 14 | 0.940 ± 0.026 | 0.01218 | 18 mo | 22 | 11 | 0.883 ± 0.047 | 0.01073 |
|  | M44C1 | 5 | Base | 22 | 15 | 0.944 ± 0.036 | 0.01119 | - | - | - | - | - |
|  | M44C2 | 4 | Base | 15 | 7 | 0.857 ± 0.065 | 0.00717 | - | - | - | - | - |
|  | M56C1 | 5 | Base | 21 | 10 | 0.814 ± 0.081 | 0.00833 | 18 mo | 20 | 11 | 0.932 ± 0.033 | 0.00757 |
|  | M65C1 | 4 | - | - | - | - | - | 18 mo | 37 | 20 | 0.874 ± 0.050 | 0.00795 |
|  | M67 | 25 | Base | 13 | 9 | 0.949 ± 0.042 | 0.02233 | - | - | - | - | - |
|  | M82C1 | 3 | Base | 15 | 10 | 0.933 ± 0.045 | 0.00767 | 6 mo | 19 | 7 | 0.877 ± 0.040 | 0.00938 |
|  | M111C1 | 5 | 6 mo | 11 | 6 | 0.855 ± 0.085 | 0.00824 | 18 mo | 32 | 14 | 0.923 ± 0.023 | 0.01232 |
| Bukoba | M7 | 34 | Base | 31 | 12 | 0.852 ± 0.051 | 0.01063 | - | - | - | - | - |
|  | M50C1 | 5 | Base | 20 | 8 | 0.858 ± 0.045 | 0.00705 | - | - | - | - | - |
|  | M57 | 30 | Base | 20 | 12 | 0.942 ± 0.032 | 0.01376 | - | - | - | - | - |
|  | M57C2 | 2 | Base | 20 | 10 | 0.905 ± 0.041 | 0.01187 | - | - | - | - | - |
|  | M65C1 | 5 | Base | 24 | 11 | 0.819 ± 0.073 | 0.00633 | - | - | - | - | - |
|  | M75 | 37 | Base | 15 | 7 | 0.810 ± 0.075 | 0.00626 | - | - | - | - | - |
| Lwanika | M23 | 41 | Base | 22 | 9 | 0.883 ± 0.042 | 0.00903 | - | - | - | - | - |
|  | M32 | 27 | Base | 44 | 13 | 0.711 ± 0.070 | 0.00496 | - | - | - | - | - |
|  | M32C1 | 4 | - | - | - | - | - | 6 mo | 21 | 9 | 0.838 ± 0.067 | 0.00626 |
|  | M43 | 24 | - | - | - | - | - | 18 mo | 9 | 7 | 0.944 ± 0.070 | 0.00978 |
|  | M43C2 | 2 | Base | 25 | 10 | 0.867 ± 0.041 | 0.00805 | - | - | - | - | - |
|  | M46C1 | 2 | Base | 19 | 12 | 0.901 ± 0.059 | 0.00903 | - | - | - | - | - |
|  | M64 | 22 | Base | 36 | 13 | 0.913 ± 0.022 | 0.01002 | - | - | - | - | - |

^a^ ID = identification code of host; ^b^ Age = host age at baseline; ^c^ *n* = number of haplotypes; ^d^ *u* = number of unique haplotypes; ^e^ *h*= haplotype diversity; ^f^ Π = nucleotide diversity. ^g^ M* = identification code for a mother host; M*C1 or M*C2 = identification code for a child host. ^h^ Base = baseline; ^I^ 6 mo = 6 months; ^j^ 12 mo = 12 months; ^k^ 18 mo = 18 months. Analysis was only carried out for infrapopulations where ≥6 parasites were successfully barcoded.
