# Supplementary material for: New Insights into the Molecular Epidemiology and Population Genetics of Schistosoma mansoni in Ugandan Pre-school Children and Mothers
Source: PLoS Negl Trop Dis. 2013 Dec 12;7(12):e2561. doi: 10.1371/journal.pntd.0002561 (PMC3861247; doi:10.1371/journal.pntd.0002561)
Supplement: Table S2 — Cox 1 diversity in infrapopulations summarised by host type, lake system and survey timepoint. (DOC) [file pntd.0002561.s002.doc]

**Table S2. *Cox*1 diversity in infrapopulations summarised by host type, lake system and survey timepoint**

| Survey | Lake | Host | N_HOSTS_^a^ | *n*^b^ | *h*^c^ | Π^d^ | Π (SS)^e^ | Π (NS)^f^ |
| --- | --- | --- | --- | --- | --- | --- | --- | --- |
| Baseline | Albert | Mothers | 14 | 167 | 0.876  (0.847-0.909) | 0.00929  (0.00680-0.01022) | 0.03821  (0.02863-0.04131) | 0.00049  (0-0.00104) |
|  |  | Children | 15 | 173 | 0.894  (0.881-0.940) | 0.00846  (0.00768-0.01029) | 0.03288  (0.02825-0.04333) | 0.00074  (0.00005-0.00111) |
|  |  | All | 29 | 340 | 0.891  (0.867-0.909) | 0.00900  (0.00769-0.00972) | 0.03575  (0.03107-0.03892) | 0.00061  (0-0.00102) |
|  | Victoria | Mothers | 8 | 188 | 0.894  (0.778-0.944) | 0.00953  (0.00584-0.01655) | 0.03403  (0.02234-0.06557) | 0.00183  (0.00020-0.00250) |
|  |  | Children | 13 | 235 | 0.901  (0.857-0.933) | 0.00805  (0.00684-0.01034) | 0.02929  (0.02433-0.03403) | 0.00134  (0.00093-0.00208) |
|  |  | All | 21 | 423 | 0.901  (0.857-0.924) | 0.00833  (0.00710-0.01035) | 0.03142  (0.02662-0.03454) | 0.00172  (0.00098-0.00190) |
|  | Both | Mothers | 22 | 355 | 0.884  (0.852-0.909) | 0.00929  (0.00791-0.01020) | 0.03599  (0.03145-0.03967) | 0.00078  (0-0.00168) |
|  |  | Children | 28 | 408 | 0.895  (0.870-0.927) | 0.00819  (0.00767-0.00925) | 0.03156  (0.02795-0.03553) | 0.00110  (0.00076-0.00133) |
|  |  | All | 50 | 763 | 0.892  (0.867-0.905) | 0.00847  (0.00773-0.00938) | 0.03599  (0.03041-0.03768) | 0.00095  (0.00067-0.00112) |
| 6 months | Albert | Mothers | 6 | 100 | 0.929  (0.863-0.935) | 0.00813  (0.00692-0.00919) | 0.03054  (0.02703-0.03425) | 0.00090  (0.00035-0.00263) |
|  |  | Children | 7 | 119 | 0.830  (0.776-0.919) | 0.00777  (0.00479-0.00984) | 0.03271  (0.01895-0.03987) | 0.00028  (0-0.00116) |
|  |  | All | 13 | 219 | 0.906  (0.815-0.929) | 0.00799  (0.00734-0.00887) | 0.03091  (0.02782-0.03397) | 0.00061  (0.00011-0.00123) |
|  | Victoria | Children | 4 | 68 | 0.847  (0.735-0.877)* | 0.00725  (0.00602-0.00938)* | 0.02533  (0.01237-0.03071)* | 0.00221  (0.00153-0.00400)* |
|  | Both | Children | 11 | 187 | 0.838  (0.784-0.885) | 0.00777  (0.00619-0.00868) | 0.02918  (0.01969-0.03390) | 0.00078  (0-0.00199) |
|  |  | All | 17 | 287 | 0.857  (0.830-0.924) | 0.00799  (0.00725-0.00840) | 0.03016  (0.02707-0.03271) | 0.00078  (0.00032-0.00153) |
| 12 months | Albert | Mothers | 1 | 12 | 0.985 | 0.00964 | 0.03695 | 0.00112 |
|  |  | Children | 5 | 90 | 0.872  (0.828-0.932)* | 0.00882  (0.00729-0.01009)* | 0.03556  (0.02469-0.03921)* | 0.00051  (0-0.00114)* |
|  |  | All | 6 | 102 | 0.891  (0.831-0.980) | 0.00887  (0.00734-0.01005) | 0.03626  (0.02549-0.03915) | 0.00735  (0-0.00114) |
| 18 months | Victoria | Mothers | 2 | 15 | 0.972  (0.944-1.000)* | 0.00913  (0.00847-0.00978)* | 0.03200  (0.02480-0.03919)* | 0.00204  (0.00074-0.00334)* |
|  |  | Children | 5 | 122 | 0.923  (0.874-0.932)* | 0.01073  (0.00757-0.01232)* | 0.03667  (0.02660-0.04482)* | 0.00175  (0.00149-0.00317)* |
|  |  | All | 7 | 137 | 0.927  (0.877-0.982) | 0.00978  (0.00769-0.01192) | 0.03667  (0.02537-0.04355) | 0.00175  (0.00098-0.00329) |

^a^ N_HOSTS_ = number of hosts; ^b^ *n* = number of samples; ^c^ *h*= median haplotype diversity (95% confidence interval); ^e^ Π = median nucleotide diversity (95% confidence interval); ^f^ Π (SS) = median nucleotide diversity at synonymous sites (95% confidence interval); ^g^ Π (NS) = median nucleotide diversity at non-synonymous sites (95% confidence interval). * Lower and upper confidence intervals held at minimum and maximum of samples. Analysis was only carried out for infrapopulations where ≥6 parasites were successfully barcoded.
