# Supplementary material for: New Insights into the Molecular Epidemiology and Population Genetics of Schistosoma mansoni in Ugandan Pre-school Children and Mothers
Source: PLoS Negl Trop Dis. 2013 Dec 12;7(12):e2561. doi: 10.1371/journal.pntd.0002561 (PMC3861247; doi:10.1371/journal.pntd.0002561)
Supplement: Table S3 — AMOVA results showing little evidence of genetic differentiation between villages at Lake Albert or Lake Victoria at baseline. (DOC) [file pntd.0002561.s003.doc]

**Table S3. AMOVA results showing little evidence of genetic differentiation between villages at Lake Albert or Lake Victoria at baseline**

|  | Lake Albert | | | | | Lake Victoria | | | | |
| --- | --- | --- | --- | --- | --- | --- | --- | --- | --- | --- |
| Source of  variation | Sum of squares | Variance  components | % of  variation | Φ_ST_^a^ | *P* value^b^ | Sum of squares | Variance  components | % of  variation | Φ_ST_ | *P* value |
| Between villages | 3.8 | -0.00009 | -0.0 | 0.0000 | 0.401 | 6.3 | -0.002 | -0.09 | 0.0000 | 0.642 |
| Among hosts  within villages | 77.2 | 0.012 | 0.7 | 0.0068 | 0.272 | 69.7 | 0.067 | 3.6 | 0.0355 | 0.0002 |
| Within hosts | 572.6 | 1.740 | 99.3 | 0.0069 | 0.297 | 738.0 | 1.791 | 90.5 | 0.0363 | <0.0001 |

^a^ Φ_ST_ estimator of genetic differentiation; ^b^ *P* value from permutation test of genetic differentiation (10000 permutations).
